# Gene Expression of GABA_A_ Receptor Subunits and Association with Patient Survival in Glioma

**DOI:** 10.3390/brainsci14030275

**Published:** 2024-03-14

**Authors:** Rafael Badalotti, Matheus Dalmolin, Osvaldo Malafaia, Jurandir M. Ribas Filho, Rafael Roesler, Marcelo A. C. Fernandes, Gustavo R. Isolan

**Affiliations:** 1 Graduate Program in Principles of Surgery, Mackenzie Evangelical University, Curitiba 80730-000, Brazilribas6015@gmail.com (J.M.R.F.); 2 The Center for Advanced Neurology and Neurosurgery (CEANNE), Porto Alegre 90560-010, Brazil; 3 InovAI Lab, nPITI/IMD, Federal University of Rio Grande do Norte, Natal 59078-970, Brazil; matheusdalmolinrs@gmail.com (M.D.); mfernandes@dca.ufm.br (M.A.C.F.); 4 Bioinformatics Multidisciplinary Environment (BioME), Federal University of Rio Grande do Norte, Natal 59078-970, Brazil; 5 Department of Pharmacology, Institute for Basic Health Sciences, Federal University of Rio Grande do Sul, Porto Alegre 90035-003, Brazil; 6 Cancer and Neurobiology Laboratory, Experimental Research Center, Clinical Hospital (CPE-HCPA), Federal University of Rio Grande do Sul, Porto Alegre 90035-003, Brazil; 7 National Science and Technology Institute for Children’s Cancer Biology and Pediatric Oncology—INCT BioOncoPed, Porto Alegre 90035-003, Brazil; 8 Department of Computer Engineering and Automation, Federal University of Rio Grande do Norte, Natal 59078-970, Brazil; 9 Spalt Therapeutics, Porto Alegre 90560-010, Brazil

**Keywords:** GABA_A_ receptor subunit gene, GABA_A_ receptor, glioma, glioblastoma, brain tumor

## Abstract

Rapid neuronal inhibition in the brain is mediated by γ-aminobutyric acid (GABA) activation of GABA_A_ receptors. The *GABRA5* gene, which encodes the α5 subunit of the GABA_A_ receptor, has been implicated in an aggressive subgroup of medulloblastoma (MB), a type of pediatric brain tumor. However, the possible role of GABA_A_ receptor subunits in glioma remains poorly understood. Here, we examined the expression of genes encoding GABA_A_ receptor subunits in different types of glioma, and its possible association with patient prognosis assessed by overall survival (OS). Data were obtained from the French and The Cancer Genome Atlas Brain Lower Grade Glioma (TCGA-LGG) datasets and analyzed for expression of GABA_A_ receptor subunit genes. OS was calculated using the Kaplan–Meier estimate. We found that genes *GABRA2*, *GABRA3*, *GABRB3*, *GABRG1*, and *GABRG2* showed a significant association with OS, with higher gene expression indicating better prognosis. In patients with GBM, high expression of *GABRA2* was associated with shorter OS, whereas, in contrast, higher levels of *GABRB3* were associated with better prognosis indicated by longer OS. In patients with lower grade gliomas, *GABRA3*, *GABRB3*, *GABRG1*, and *GABRG2*, were associated with longer OS. High *GABRB3* expression was related to longer survival when low grade glioma types were analyzed separately. Our results suggest an overall association between higher expression of most genes encoding GABA_A_ receptor subunits and better prognosis in different types of glioma. Our findings support the possibility that down-regulation of GABA_A_ receptors in glioma contributes to promoting tumor progression by reducing negative inhibition. These findings might contribute to further evaluation of GABA_A_ receptors as a therapeutic target in glioma.

## 1. Introduction

Gliomas are the most common malignant brain tumors, comprising about 80 percent of central nervous system (CNS) cancers in adults. Glioma types are broadly classified into astrocytoma, oligodendroglioma and glioblastoma (GBM) [1]. According to current World Health Organization (WHO) classification, glioma types spam from the least aggressive grade 1 to the most aggressive grade 4 tumors, based on a range of cellular, histological, and pathological features, including cellular morphological changes and proliferative capacity [2]. Grade 1 and grade 2 gliomas are considered low-grade gliomas (LGGs), which show relatively few cellular alterations, or grade 2 gliomas, which show cellular atypia. Low-grade tumors include diffuse astrocytomas, pilomyxoid astrocytomas, pilocytic astrocytomas, oligodendrogliomas, and oligoastrocytomas, among others [3]. The most prevalent and lethal primary glioma type is grade 4 GBM, which accounts for about half of newly diagnosed gliomas. GBM can be classified into three groups depending on the status of the isocitrate dehydrogenase (*IDH*) gene: *IDH* wild-type GBM, which represents about 90% of cases, mutated *IDH*, or not specified GBM (NOS, unevaluated status). Tumors with an *IDH* mutation arise from lower-grade gliomas [4]. Increasing evidence indicates that the cells of origin of GBM are likely neural stem cells in the subventricular zone (SVZ) of the adult human brain. The SVZ is a layer between the lateral ventricle, corpus callosum, and striatum, which has the largest number of neural stem cells in the brain [5,6,7]. These cells can contain many of the driver mutations that give rise to GBM, share molecular features with GBM cells, and display migratory patterns from the SVZ to the tumor. In addition, key genetic mutations in GBM are associated with genes that regulate neuronal function in the SVZ [8,9,10,11,12].

Surgical treatment stands as the main therapeutic intervention in the management of gliomas, including GBM. The extent of GBM tumor surgical resection strongly influences the prognosis so that incomplete resections result in earlier worsening in neurological function, and, for recurrent GBM, repeated surgical resection is usually recommended [13]. In addition to surgery, multimodal therapy for GBM included radiotherapy and chemotherapy with temozolomide. Despite advances in therapy, prognosis remains dismal, with most patients having a median overall survival of 12–15 months [4,14]. Thus, there is an urgent need for novel biomarkers and molecularly targeted therapeutics that improve the diagnostic and pharmacological treatment of GBM [15,16].

Neurotransmitters and their receptors in tumor cells or the tumor microenvironment are increasingly recognized as regulators of cancer cells and neuron–tumor interactions that contribute to tumor progression [17,18]. The major inhibitory neurotransmitter in the CNS is γ-aminobutyric acid (GABA). Rapid neuronal inhibition is mediated by GABA-induced activation of the GABA_A_ type of receptor, which forms a ligand-gated chloride (Cl^−^) ion channel. Upon GABA binding to the receptor, Cl^−^ influx leads to membrane hyperpolarization and consequently neuronal inhibition. In addition to mediating fast neuronal inhibition in the adult brain, GABA and its receptors regulate CNS development [19], proliferation and differentiation of neural stem cells and neuronal progenitors [20,21,22], and adult neurogenesis [20,23,24,25]. As discussed above, neural stem cells in the SVZ are proposed as cells of origin in GBM [8,9,10,11,12]. GABA has been shown to depolarize neuronal progenitors in the SVZ through activation of GABA_A_ receptors [26]. GABA_A_ activation increases cellular calcium in neural progenitors and astrocyte-like cells in the SVZ [27,28], and modulates maturation, differentiation, and migration of SVZ neuronal progenitors [29,30].

GABA_A_ receptors consist of a combination of five proteins drawn from a repertoire of 19 subunits (α1-6, β1-3, γ1-3, δ, ε, θ, π, ρ1-3). Most functional GABA_A_ receptors consist of two α, two β and one γ or δ subunit [31,32,33]. The *GABRA5* gene encodes the α5 subunit of the GABA_A_ receptor, and mutations in *GABRA5* have been associated with epilepsy [34,35]. In brain tumors, GABA transmission has been proposed to influence seizures associated with GBM [36]. Also, increased levels of *GABRA5* were described in the most aggressive molecular subgroup, namely Group 3, of medulloblastoma (MB), the main type of malignant brain cancer afflicting children. Experimental activation of GABA_A_ receptors containing the α5-subunit can reduce cell survival in MB [37]. However, it remains unknown how GABA_A_ receptors containing different subunit repertoires impact in GBM tumor cells influences tumor progression and clinical prognosis. Here, we examined transcript levels of GABA_A_ receptor subunits in different types of glioma and their possible implications for patient survival.

## 2. Materials and Methods

### 2.1. Glioma Tumor and Patient Data

Gene expression data used in this study were acquired from the Gene Expression Omnibus (GEO) [PMC4944384]. The French dataset (GSE16011, GPL570 Affymetrix Human Genome U133 Plus 2.0 Array) includes expression information from primary glioma tumor biopsies and 8 non-tumoral neural tissue samples which were used as controls [PMID: 19920198].

Normalization of raw microarray data was performed using the Robust Multichip Average (RMA) method, and quality control was conducted through Affy Bioconductor/R [PMID: 14960456]. GPL570 annotations were downloaded from the database: https://www.ncbi.nlm.nih.gov/geo/query/acc.cgi?acc=GPL570. Clinical information on patients from the French cohort was obtained through the ‘geoquery’ package and the original article describing processing of these data.

We also examined data from The Cancer Genome Atlas Brain Lower Grade Glioma cohort (TCGA-LGG) [38,39]. Processed and normalized expression data were obtained from the cBioPortal. Five hundred and thirteen primary tumor samples were used in our analysis. Clinical information about patients in the TCGA-LGG cohort was acquired through the cBioPortal.

### 2.2. Statistics

Nineteen GABA_A_ receptor subunits are known (PMC8380214). The French dataset contains includes 18 genes encoding GABA_A_ receptor subunits. These 18 genes are represented by 36 probes_id (GPL570). We investigated the relationship between gene expression level in the 36 probes_id and overall survival (OS) of glioma patients. Eight control samples and 12 tumor samples in the French dataset that lacked information about patient status (‘alive’ or ‘dead’) were excluded from our analysis, resulting in a total of 266 analyzed samples. Characteristics of patients in both the French and TCGA-LGG datasets have been previously described [38,39]. We used the “Survminer” package with ‘minprop = 0.2’ to classify patients as “high” and “low” gene expression levels. Survival analysis was conducted using the “Survival” package” (version 3.5-5, https://github.com/therneau/survival). 

## 3. Results

### 3.1. GABA_A_ Receptor Genes Influencing OS in Patients with Glioma

First, OS analyses were conducted using 266 glioma samples from the French dataset. Patients were divided into two groups based on the expression level of each of the 36 probes corresponding to 18 genes that compose the GABA_A_ receptor, high or low. Eleven probes representing five genes, namely *GABRA2*, *GABRA3*, *GABRB3*, *GABRG1*, and *GABRG2*, showed a significant association with OS, with high expression indicating better prognosis (Bonferroni-adjusted *p* < 0.05). For each of the five genes, when necessary, we selected the probe with the lowest Bonferroni-adjusted *p* value and used that probe for the remaining analyses (Table 1).

### 3.2. GABRA2 and GABRB3 Genes Display Opposite Patterns of Association with OS in Patients with GBM

**Figure 1:**
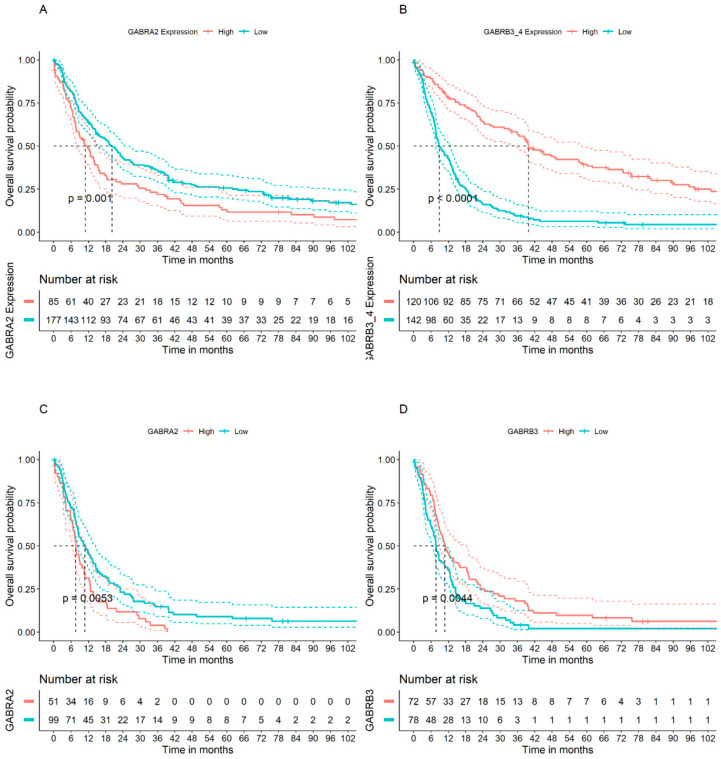
OS analysis of the genes *GABRA2* and *GABRB3* in patients from the French cohort. Results are derived from all glioma tumor types pooled together (*n* = 266) for (**A**) *GABRA2* and (**B**) *GABRB3*; and GBM only (*n* = 153) for (**C**) *GABRA2* and (**D**) *GABRB3*.

We then selected the samples within the French cohort classified as glioblastoma (GBM) (*n* = 153). Genes *GABRA2* and *GABRB3* had a Bonferroni-adjusted *p* value < 0.05 in these tumor samples (Table 2). High expression of *GABRA2* was associated with worse prognosis (Figure 1A,C), whereas, in contrast, high levels of *GABRB3* transcripts were associated with better prognosis indicated by longer OS (Figure 1B,D). It is worth highlighting that *GABRA2* was the only GABAA receptor gene associated with worse prognosis in GBM patients.

### 3.3. GABA_A_ Receptor Genes and OS in Patients with Lower Grade Glioma Types

We next analyzed glioma tumors from the TCGA-LGG cohort containing 513 samples distributed across glioma subtypes astrocytoma, oligoastrocytoma, and oligodendroglioma. Using all samples in the dataset (*n* = 513), we carried out OS analyses for *GABRA2*, *GABRA3*, *GABRB3*, *GABRG1*, and *GABRG2* genes. All genes except for *GABRA2* showed significant association with OS, where higher gene expression was related to longer OS (Bonferroni-adjusted *p* < 0.05) (Table 3).

**Figure 2:**
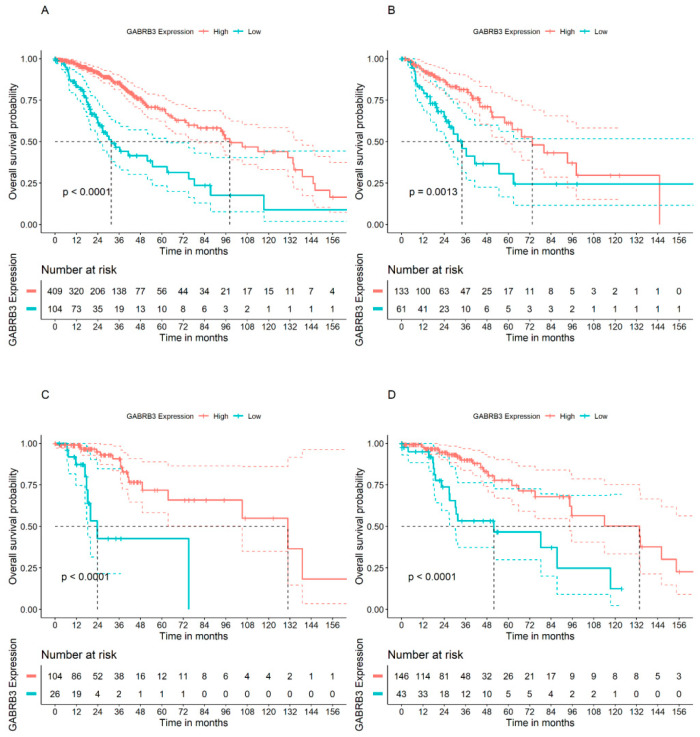
Analysis of OS in patients bearing high or low tumor levels of *GABRB3* in the TCGA-LGG cohort. (**A**) All glioma types pooled together (*n* = 513), (**B**) astrocytoma (*n* = 194), (**C**) oligoastrocytoma (*n* = 130), and (**D**) oligodendroglioma (*n* = 189).

We went on to verify whether the *GABRB3* gene, which showed significant associations with OS in GBM patients from the French cohort and also for TCGA-LGG patients when all tumor types were pooled together, would show influences on OS when lower grade tumors are analyzed separately. Higher *GABRB3* expression levels were significantly associated with OS in all glioma subtypes, namely astrocytoma, oligoastrocytoma, and oligodendroglioma (Bonferroni-adjusted *p* < 0.05) (Figure 2).

## 4. Discussion

Functional GABA_A_ receptors were initially identified in cells derived from lower grade gliomas, namely astrocytoma and oligodendroglioma, whereas GBM-derived primary cells and glioma cell lines showed no functional receptors. In tumor-derived glioma cells in acute slices or primary culture, most cells from oligodendroglioma and astrocytoma responded to GABA when responses were measured in whole-cell voltage clamp assays as inward currents under high Cl^−^ concentration. GBM-derived cells, in contrast, showed no response to GABA. The currents observed in lower grade gliomas were induced specifically by GABA through activation of GABA_A_ receptors, given that the GABA_A_ agonist muscimol mimicked the GABA responses, the benzodiazepine receptor agonist flunitrazepam augmented GABA-induced currents, a benzodiazepine inverse agonist reduced the currents, and the GABA_A_ antagonists bicuculline and picrotoxin blocked GABA-induced currents. It is also noteworthy that, in this experimental setting, GABA-elicited currents could induce either hyperpolarization or depolarization, depending on the cell tested [40]. Functional GABA_A_ receptor-activated currents in GBM cells were later demonstrated, as were findings showing that endogenous GABA continuously released by GBM cells could reduce proliferation of cells expressing progenitor and stem cells markers and negatively regulate experimental tumor growth in mouse models. Thus, shunting cellular Cl^−^ chloride ions through sustained local GABA_A_ receptor activity reduced proliferation and tumor growth and prolonged mouse survival. These results strongly suggest that increasing GABA_A_ receptor activity may inhibit GBM progression [41]. In U3047MG human GBM cells, GABA_A_ currents could be pharmacologically stimulated by etomidate, propofol, or diazepam, indicating that GABA-induced currents in GBM can be enhanced by classical GABA_A_ receptor-stimulating drugs. Expression of nRNAs for the α2, α3, α5, β1, β2, β3, δ, γ3, π, and θ GABA_A_ receptor subunits was confirmed in U3047MG cells [37,42]. Together, these findings indicate that glioma tumors of different grades can express GABA_A_ receptors capable of responding to endogenous GABA and other ligands to affect glioma progression.

Expression of mRNA for all 19 GABA_A_ subunits in human glioma (*n* = 29) and peri-tumoral tissue (*n* = 5) was previously detected. Consistently with the possibility that lower GABA_A_ receptor activity occurs in more malignant gliomas, GBM tumors showed reduced subunit levels compared to lower grade gliomas, except for the θ subunit. Expression was also found in peritumoral tissue. A consistent co-expression of ρ2 and θ subunits occurred in both astrocytomas and oligodendroglial tumors. Expression of the ρ2 subunit but not the θ subunit was shown by Kaplan–Meier analysis and Cox proportional hazards modeling to be an independent predictor of better survival in patients with astrocytomas, together with other prognostic factors [43].

Isocitrate dehydrogenase (IDH) enzymes, encoded by *IDH* genes, regulate cellular metabolism and homeostasis by catalyzing the oxidative decarboxylation of isocitrate. Accumulating evidence shows that *IDH* genes can be mutated in many human malignant cancers, gliomas, and these mutations can impact oncogenesis, tumor progression, and clinical outcome. In gliomas, *IDH* mutation-associated abnormal changes in cancer cell metabolism, gene expression profile and chromatin structure can lead to disruptions in normal epigenetic programming and, ultimately, resistance to therapy. Thus, increasing research efforts focus on therapeutic strategies designed to specifically target *IDH*-mutant gliomas [44,45,46,47]. Some *IDH1* mutations in glioma are proposed as prognostic markers, with patients bearing mutated tumors showing improved survival [48]. Analysis of tumors from TCGA showed eight subunit genes significantly expressed in *IDH* wild-type compared with *IDH*-mutated tumors. Higher expression of the *GABRD* gene, which encodes the GABA_A_ receptor δ subunit, was independently associated with longer patient OS in *IDH* wild-type LGGs. *GABRD* expression was negatively correlated with the extent of tumor infiltration by macrophages. These results suggest that *GABRD* may be a potential independent prognostic marker in patients with *IDH* wild-type LGG [49]. Our findings indicating that expression of most GABA_A_ receptor subunit genes is reduced in patients with longer OS may be considered consistent with previous evidence that GABA_A_ receptors can act as inhibitors of glioma growth [41] that display lower expression as glioma grade increases [43].

Also, consistently with an inhibitory role for GABA_A_ receptors in brain tumors, receptor pharmacological stimulation with benzodiazepine derivatives promotes cell death in experimental MB [50]. Current consensus classifies MB tumors into four molecular subgroups, namely wingless activated (WNT), sonic hedgehog (SHH), Group 3, and Group 4, with Group 3 and Group 4 tumors being particularly aggressive [51,52]. *GABRA5* and the α5 subunit are found and contribute to the assembly of functional GABA_A_ receptors in patient-derived Group 3 MB cells and tumor tissue. In addition, a benzodiazepine preferentially targeting α5-GABA_A_ hinders Group 3’s MB cell viability [37] with greater potency than standard-of-care chemotherapy used to treat MB patients [53]. Stimulation of GABA_A_ receptors containing the α5 subunit with a selective agonist reduces cell survival through a mechanism involving membrane depolarization and apoptosis induction [37], highlighting the potential of the α5-GABA_A_ receptor as a therapeutic target [54]. There is a significant correlation between expression of *GABRA5* and the *MYC* oncogene in a subset of Group 3 and WNT MB tumors, and the same study indicated *GABRA5* expression as a possible diagnostic marker for Group 3 MB [50].

## 5. Conclusions

In summary, the present study is the first to characterize gene expression of the different protein subunits composing the GABA_A_ receptor in distinct types of glioma, showing that most genes are associated with better prognosis assessed by patient OS, which is consistent with an inhibitory role of GABA in glioma growth. In light of the evidence reviewed above, our findings raise the possibility that glioma tumors show a down-regulation of GABA_A_ receptors as a mechanism to stimulate tumor growth by reducing inhibitory modulation. It should be pointed out, however, that additional functional studies are required to further validate this hypothesis, given that our findings are limited to gene expression and do not confirm that GABA_A_ are directly implicated in determining patient outcomes. Drugs that act by stimulating GABA_A_ receptors should be further investigated as targeted therapies for glioma.

## Figures and Tables

**Table 1:** Summary of patient OS analysis results conducted for all 36 probes corresponding to 18 genes that encode GABA_A_ subunit proteins in the French dataset.

Probe	Subunit	Gene	*p* Value	Adjusted *p*
206678_at	GABA_A_ receptor, alpha 1	*GABRA1*	1.72 × 10^−1^	1
244118_at	GABA_A_ receptor, alpha 1	*GABRA1*	1.68 × 10^−1^	1
1554308_s_at	GABA_A_ receptor, alpha 2	*GABRA2*	1.00 × 10^−3^	3.60 × 10^−2^
207014_at	GABA_A_ receptor, alpha 2	*GABRA2*	1.29 × 10^−2^	4.64 × 10^−1^
216039_at	GABA_A_ receptor, alpha 2	*GABRA2*	1.24 × 10^−3^	4.45 × 10^−2^
207210_at	GABA_A_ receptor, alpha 3	*GABRA3*	1.05 × 10^−7^	3.78 × 10^−6^
208463_at	GABA_A_ receptor, alpha 4	*GABRA4*	2.99 × 10^−1^	1
233437_at	GABA_A_ receptor, alpha 4	*GABRA4*	1.83 × 10^−1^	1
206456_at	GABA_A_ receptor, alpha 5	*GABRA5*	7.45 × 10^−2^	1
215531_s_at	GABA_A_ receptor, alpha 5	*GABRA5*	2.69 × 10^−1^	1
217280_x_at	GABA_A_ receptor, alpha 5	*GABRA5*	1.16 × 10^−1^	1
207182_at	GABA_A_ receptor, alpha 6	*GABRA6*	1.42 × 10^−2^	5.10 × 10^−1^
1557256_a_at	GABA_A_ receptor, beta 1	*GABRB1*	0.01	0.48
207010_at	GABA_A_ receptor, beta 1	*GABRB1*	2.03 × 10^−2^	7.31 × 10^−1^
1557122_s_at	GABA_A_ receptor, beta 2	*GABRB2*	9.31 × 10^−3^	3.35 × 10^−1^
207352_s_at	GABA_A_ receptor, beta 2	*GABRB2*	3.49 × 10^−1^	1
242344_at	GABA_A_ receptor, beta 2	*GABRB2*	3.62 × 10^−2^	1
1569689_s_at	GABA_A_ receptor, beta 3	*GABRB3*	1.51 × 10^−2^	5.45 × 10^−1^
205850_s_at	GABA_A_ receptor, beta 3	*GABRB3*	2.43 × 10^−13^	8.74 × 10^−12^
227690_at	GABA_A_ receptor, beta 3	*GABRB3*	1.21 × 10^−14^	4.36 × 10^13^
227830_at	GABA_A_ receptor, beta 3	*GABRB3*	5.55 × 10^−16^	2.00 × 10^−14^
229724_at	GABA_A_ receptor, beta 3	*GABRB3*	0	0
208457_at	GABA_A_ receptor, delta	*GABRD*	2.04 × 10^−2^	7.35 × 10^−1^
230255_at	GABA_A_ receptor, delta	*GABRD*	1.34 × 10^−1^	1
1552943_at	GABA_A_ receptor, gamma 1	*GABRG1*	8.35 × 10^−6^	3.01 × 10^−4^
241805_at	GABA_A_ receptor, gamma 1	*GABRG1*	1.43 × 10^−6^	5.16 × 10^−5^
1568612_at	GABA_A_ receptor, gamma 2	*GABRG2*	1.63 × 10^−6^	5.88 × 10^−5^
206849_at	GABA_A_ receptor, gamma 2	*GABRG2*	7.95 × 10^−8^	2.86 × 10^−6^
1555517_at	GABA_A_ receptor, gamma 3	*GABRG3*	1.44 × 10^−2^	5.18 × 10^−1^
216895_at	GABA_A_ receptor, gamma 3	*GABRG3*	1.65 × 10^−1^	1
205044_at	GABA_A_ receptor, pi	*GABRP*	2.78 × 10^−1^	1
220886_at	GABA_A_ receptor, theta	*GABRQ*	3.44 × 10^−1^	1
238123_at	GABA_A_ receptor, theta	*GABRQ*	4.06 × 10^−1^	1
206525_at	GABA_A_ receptor, rho 1	*GABRR1*	4.44 × 10^−3^	1.60 × 10^−1^
208217_at	GABA_A_ receptor, rho 2	*GABRR2*	4.71 × 10^−2^	1
234410_at	GABA_A_ receptor, rho 3	*GABRR3*	1.34 × 10^−2^	4.84 × 10^−1^
206678_at	GABA_A_ receptor, alpha 1	*GABRA1*	1.72 × 10^−1^	1

**Table 2:** Summary of the patient OS analysis results carried for five GABA_A_ receptor subunit genes in GBM patients from the French cohort.

Probe	Subunit	Gene	*p* Value	Adjusted *p*
1554308_s_at	GABA_A_ receptor, alpha 2	*GABRA2*	5.34 × 10^−3^	2.67 × 10^−2^
207210_at	GABA_A_ receptor, alpha 3	*GABRA3*	2.02 × 10^−2^	1.01 × 10^−1^
229724_at	GABA_A_ receptor, beta 3	*GABRB3*	4.39 × 10^−3^	2.19 × 10^−2^
206849_at	GABA_A_ receptor, gamma 1	*GABRG1*	1.57 × 10^−1^	7.83 × 10^−1^
241805_at	GABA_A_ receptor, gamma 2	*GABRG2*	8.95 × 10^−2^	4.48 × 10^−1^

**Table 3:** Summary of the patient OS analysis results carried for GABA_A_ receptor subunit genes in lower grade glioma patients from the TCGA-LGG cohort.

Subunit	Gene	*p* Value	Adjusted *p*
GABA_A_ receptor, alpha 2	*GABRA2*	5.37 × 10^−2^	2.69 × 10^−1^
GABA_A_ receptor, alpha 3	*GABRA3*	6.25 × 10^−14^	3.13 × 10^−13^
GABA_A_ receptor, beta 3	*GABRB3*	1.63 × 10^−11^	8.13 × 10^−11^
GABA_A_ receptor, gamma 1	*GABRG1*	4.13 × 10^−7^	2.07 × 10^−6^
GABA_A_ receptor, gamma 2	*GABRG2*	1.96 × 10^−5^	9.78 × 10^−5^

## Data Availability

All data were generated and analyzed during this study are based on publicly available datasets and softwares, as described in the article.
